# Who is informed and who uninformed? Addressing the legal barriers to progress in dementia research and care

**DOI:** 10.1186/s13584-018-0279-z

**Published:** 2019-02-20

**Authors:** Jiska Cohen-Mansfield

**Affiliations:** 1 Department of Health Promotion, School of Public Health, Sackler Faculty of Medicine, Tel-Aviv University, P.O.B. 39040, Ramat Aviv Tel-Aviv, Tel-Aviv, Israel; 2 Minerva Center for the Interdisciplinary Study of End of Life, Tel Aviv University, P.O.B. 39040, Ramat Aviv Tel-Aviv, Tel-Aviv, Israel; 3 The Herczeg Institute on Aging, Tel-Aviv University, P.O.B. 39040, Ramat Aviv Tel-Aviv, Tel-Aviv, Israel

**Keywords:** Dementia, Informed consent, Ethics, Human subjects research

## Abstract

Conduct of research is an essential tool for the evaluation and improvement of health services. In Israel, research on persons with dementia is very limited, with the largest portion of such research involving a few surveys and examining risk factors for dementia. Very few studies describe clinical research, and those that do either include participants at early stages of dementia, or rely completely on caregivers’ perceptions and experiences, often without reference to any individual with dementia. This dearth of research is due, to a substantial extent, to Ministry of Health regulations which do not permit family proxy consent for research involving persons with dementia. Alternative models for regulation of consent for research exist in other countries, including the U.S., and these allow for proxy consent under certain conditions. This paper presents such a model and its underlying ethical principles. It contends that the current state of affairs, which stands in the way of clinical research concerning persons with advanced dementia, is contrary to the interests of such persons, their caregivers, and Israeli society. Therefore, this paper calls for a change in the present regulations and/or law in the cause of advancing knowledge and improving care for persons with dementia.

## Background

The term “dementia” refers to a group of clinical conditions characterized by a cognitive decline that evolves into functional decline which interferes with the ability to perform activities of daily living, including mobility and ability to take care of oneself in terms of dressing, eating, and other functions. The deterioration of cognitive abilities compromises awareness, memory, and the ability to communicate effectively.

Research concerning persons with dementia (PwDs) is a necessary condition for providing good care. Because of their multiple complex needs, PwDs are often dependent on their environment - caregivers and the physical and social environment - for their needs and pleasures, and for the avoidance of pain, both physical and emotional. These circumstances render PwDs a vulnerable population, thereby making it imperative to conduct empirical research, especially research using persons from this population. Over the past 30 years there has been a significant increase in such research, which has brought about important insights in understanding PwDs and the care they need. The goal of this paper is to examine both the state of dementia research in Israel and the barriers which impede progress, while also comparing Israeli regulations to those in the United States and other countries in which such research is conducted.

### The need for clinical research in dementia

Clinical research in dementia includes studies that describe the types and prevalence of problems that arise in caring for PwDs; investigate the etiology of those problems and test solutions; study the perspective of PwDs concerning the quality of their lives, including their needs and preferences; develop care alternatives and innovations in the provision of care, including pain and discomfort care, activities of daily living (ADL) care, individual and group activities, and methods for decreasing feelings such as loneliness and anxiety; test the efficacy and risks of technologies for the care of PwDs; study the needs of formal and family caregivers and means of improving communication between them and the PwDs for whom they care. Such research is a prerequisite for improving the quality of life and quality of care for PwDs, and for optimizing person-centered care. Clinical research includes behavioral and non-pharmacological research as well as biological, devise testing and pharmaceutical research.

Examples of groundbreaking clinical research with PwDs abound, and this has changed guidelines for practice in areas such as treating behavior problems like agitation, understanding psychotic symptoms, and creating a consensus concerning the need to eliminate the use of physical and chemical restraints in the treatment of this population. Most of this pioneering research has been conducted in the United States, Great Britain, and Scandinavia.

In contrast, in Israel, such research is extremely rare, and answers to even the most fundamental questions, such as the prevalence of behavioral problems or of the use of physical restraints with PwDs, remain unknown. The limited available research is generally indirect. One study, which relied on interviews with physicians in nursing homes, found that there were higher rates of physical restraints use by physicians in Israel (22%) than in the U.S. (4%). In addition, the vast majority (92.5%) of interviewed physicians reported using psychotropic medications for behavioral problems in the last patient they treated. Physicians also reported insufficient familiarity with nonpharmacological interventions [1]. Another study found inadequate quality of care of persons with advanced dementia living in the community [2]. Yet, these studies only serve to highlight the need for further and more in-depth research, as they do not provide true prevalence, etiology, or solution. From the point of view of health policy, this means that data to inform policy are unavailable. In fact, the lack of appropriate clinical research renders all parties uninformed — PwDs, family caregivers, formal caregivers, facility administrators, policy makers, and decision makers at all levels of jurisdiction.

The scarcity of clinical research in Israel for the care of PwDs leaves multiple basic questions unanswered. What is the standard of care? What are the most urgent priorities for investment in improvement? How does our knowledge and practice compare with that of other countries? Without research, decision makers are deprived of the data necessary to achieve progress in care. Without data, informed, rational decision-making is not possible. Instead, choices about program adoption and implementation are subject to the wishes or interests of those who implement them, and therefore, decisions are likely to be biased and inadequate.

Years of research suggest that not only is clinical research likely to result in benefits to the general population, the research participants themselves are likely to benefit from the process of research. During studies, the PwDs frequently receive personal attention which is generally a scarce commodity in their usual care. Moreover, research usually involves a medical record review, which sometimes leads to additional attention being directed to the care plan or to the detection of errors in practice. The research process is often beneficial to caregivers as well, both formal and informal. Some caregivers have expressed appreciation for the opportunity to participate in research interviews, as the interview questions often prompted them to focus on a systematic examination of aspects of the experience of the persons under their care, aspects which otherwise might have been neglected in the rush to provide care. This process, thus draws attention to the experience and plight of caregivers. When interviewed, some nursing assistants have noted that the interview served as the first time in which their role was respected and their perceptions elicited regarding the state and needs of the care recipient.

### Research on persons with dementia in Israel

A PubMed search of research articles using the MeSH term of “dementia,” and the word ‘Israel’ appearing in the title and/or abstract, with a publication date between the years of 2000 and 2017, and limited to humans, resulted in 76 papers. Of these, five studies had not been conducted in Israel, nor authored by Israeli authors, leaving 71 papers, of which 12 were review or opinion papers. The remaining 59 papers, published over a span of close to 18 years, are summarized in Table 1. The largest subgroup of these includes 18 epidemiological papers, most of which represent analyses of two databases – the follow-up of the Israel Ischemic Heart Disease study and a study of several Arab villages in the north of Israel. This latter study also provided the data for some of the nine papers focusing on genetic markers of dementia. A further group of papers studied attitudes towards PwDs using methods such as vignettes presented to physicians [2] or to lay persons [3] or questionnaires presented to nurses and social workers [4]. Very few studies focused on clinical care, its quality, or the lived experience of PwDs that should inform clinical care (See Table 1). This last group of studies were usually based on interviews with informal or formal caregivers [5–8] or on medical records review [9, 10]. Only one study included a small clinical trial with eight patients [11] with mild cognitive impairment. An inspection of the studies summarized in Table 1 reveals that, not only is the number of studies on PwDs small, but the majority of these were conducted by just a few research groups and covered a very limited range of topics. Furthermore, the clinical studies typically did not directly involve PwDs; when studies examined care, they tended to ask caregivers about how they provided care, rather than asking how specific PwDs were treated.

**Table 1:** Research types in Israel according to Pubmed search 2001–2017

Type of research	Number of Papers	Main research group	References
Reviews or opinion papers	12	Multiple researchers	
Surveys	Total: 18		
Survey - Israel Ischemic Heart Disease study	9	Goldbourt, Beeri, Ravona-Springer	[37–45]
Survey of Arab population	7	Bowirrat, Mizrahi, Friedland, Korczyn, Inzelberg	[46–52]
Other surveys	2		[53, 54]
Genetic risk factors	9	Friedland, Korczyn, Inzelberg	[55–63]
Attitudes, knowledge, and behaviors towards PwD	6	Werner	[2–4, 64–66]
Psychological states of PwD: Psychotic symptoms, self-identity	5	Cohen-Mansfield, Golander	[5, 67–70]
Mobility patterns and use of GPS	3	Auslander, Shoval	[71–73]
Quality of care, feeding tubes	8		[1, 6–10, 74, 75]
Costs of care	2		[76, 77]
Care by migrant workers	2	Ayalon	[78, 79]
International study on use of psychotropic drugs	2	No Israeli author	[80, 81]
Other	4		
Total	71		

The studies presented in Table 1 are obviously limited by the search method. One study not identified by this search was a longitudinal survey of health and function of people living in Jerusalem. This reported that at age 85, 23% of participants had cognitive impairment (with 10% having a MMSE of less than 10 and another 13.3% scoring between 18 and 23), but the study did not classify participants in terms of dementia [12]. In cases where participants were extremely frail or had dementia, consent was obtained from legal guardians who served as proxy informants. This, however, would not be feasible for most clinical studies on persons with advanced dementia, as most do not have a legal guardian, nor is it necessarily in their best interest to have a guardian [13, 14].

### Regulation as a major barrier to clinical research of dementia in Israel

Perhaps the most significant barrier to clinical research of dementia experience and care in Israel is found in Regulatory Procedure 14, which was promulgated in 2016 by the Ministry of Health. [15]. These regulations are a follow-up to the 1962 Law of Legal Capacity and Guardianship, which did not mention research and focused primarily on the status of minors and guardianship [16]. Section 2.2 of Regulatory Procedure 14 states that “A family member who has not been appointed as legal guardian is not allowed to consent for a participant” [who is unable to provide consent] [15]. Given that most persons with dementia do not have a legal guardian, the guidelines preclude clinical research, except with persons who have very mild early stage dementia.

Regulatory Procedure 14 is in accord with existing legislation, e.g., the Nation’s Health Act [17], the Patient’s Rights Law [18] and the Law of Legal Capacity and Guardianship [19] in that those laws do not empower relatives to provide consent for a legally incompetent adult (though they do not forbid it). However, in clinical practice this is obviously a common practice despite its lack of basis in Israeli law. When a medical decision must be made for a person with advanced dementia, family members are consulted and they do make decisions for the legally incompetent patient, but this practice does not extend to decisions regarding participation in research.

While the Law of Legal Capacity and Guardianship [19] empowers legally competent adults to execute a durable power of attorney form in which they can appoint a relative to make decisions concerning future medical treatments, the vast majority of the population does not make use of this option, as is true in other countries in which the option exists.

In practice, the regulations are tantamount to a ban on research of more advanced dementia. This indiscriminate limitation poses an ethical problem because it does not afford consideration of the risks and benefits of such research, nor does it address the need to improve the lot of the very population in whose name the regulations were promulgated.

The regulations present additional restrictive conditions which may prevent the conduct of clinical research. Section [2.215] [15] provides that “[i]f the investigator has doubt concerning the ability of a participant to provide an informed consent, and the investigator knows that the participant has no legal guardian, the investigator has to get an evaluation by a psychiatrist, a geriatrician, or an expert physician in the pertinent area who is unrelated to the study.” Such a requirement places a heavy financial burden on the study. This could potentially be borne by studies sponsored by pharmaceutical companies, but not by the more common clinical studies performed by staff members at clinics or nursing homes or by students, or studies funded by the generally low research grants which may be obtained from local sources. Some of the other problematic sections of the regulations include limiting the investigator or principal investigator to physicians or dentists [15]. Although there is a provision which states, “[i]n research of data and questionnaires, a person with a lower graduate degree [(i.e., MA or MSc)] can serve as a principal investigator,” this clause effectively restricts the research to retrospective questionnaires or to chart reviews without contact with participants. What about studies of nonpharmacological interventions, such as music or group discussions? What about observation of PwDs? The regulations pertaining to these types of research seem to be the same as those of pharmacological studies, i.e., posing insurmountable barriers.

It appears that previous regulations from 1980 [20] may have allowed consent by family, but this is ambiguous because the sentence (“In case of physical or mental incapacity which render obtaining informed consent impossible, or where the person being used for research is a minor, permission from the responsible family member will be obtained instead of the one by the person to be included in research…” (page 6, item 11)) comes immediately after the section requesting consent by a formal guardian, and because permission is not considered an informed consent. Regulatory Procedure 14 [15] provides (in Appendix 5, page 76) for exemption from consent in rare cases that promise future benefit to patients with the same condition, in cases in which the inquiring committee decides that the research is very important because of anticipated significant contribution to improvement of medical care, the research procedures are not invasive, the family members have been informed and have not objected, etc., and the study is also approved by the superior (national) committee for medical trials in human subjects [19]. Requirements such as these cause researchers to either avoid the topic or restrict it to caregiver reports or record review, thereby limiting the scope and value of their studies. Yet, if these requirements are modified to authorize all ethics committees to determine the propriety of proposed studies, and to change the standard from “very important” to “important,” this may enable behavioral research on care for persons with dementia.

### Other barriers to clinical research with persons with dementia in Israel and elsewhere

Unlike the U.S. or Great Britain, where the governments have designated substantial funds for research with PwDs, no such funding has been provided in Israel. Thus, lack of funds is another major barrier to clinical research. This can be considered part of a vicious cycle, i.e., if regulation stands in the way of such research, why provide funds for it? Generally, there are two main sources for research funding: pharmaceutical companies, which initiate trials based on the anticipated return on investment, and public funds. Generally, only public funds (mostly governmental agencies, but sometimes charitable foundations) finance research aimed at improvement in processes of care.

Even in countries which recognize the need for a more flexible approach to regulation of dementia research, consent can be difficult to obtain [21, 22]. Rates of consent have often been low, especially in pharmacological studies, since those studies often require participants to be relatively healthy, whereas PwDs characteristically suffer from multiple chronic conditions and often from acute ones as well. Universal difficulties in obtaining consent for research with PwDs may arise from process issues such as lack of availability of a close family member who might provide consent [17] – even in jurisdictions in which it is legal to obtain consent from relatives. Determining the capacity of PwDs to provide consent can also be challenging, notwithstanding the suggestion of multiple approaches to this challenge [21, 22]. In studies of care of persons with more advanced levels of dementia, researchers may encounter relatives of PwDs who refuse to provide consent out of a sense of resignation or despair and of certainty that no study will help their loved one. Alternately, in studies of care of persons in the early to moderate stages of dementia, investigators may assume mistakenly that the PwDs cannot execute an informed consent document because of memory problems or the inability to pass a comprehension test. However, many such PwDs may understand what the study entails, can decide whether they would like to participate, and can make their preferences known. An insufficient recognition of the remaining abilities of such PwDs may deprive them of the opportunity to make their own decision and to participate in research.

### Informed consent and assent

The dilemmas which arise during the conduct of research with PwDs are discussed in several papers, some of which suggest protocols for handling issues of consent and assent [21, 23–25]. Informed consent is an agreement, either verbal or in written form, to participate in research. It needs to be (1) voluntary, i.e., giving the person the choice to consent or to decline, (2) informed, i.e., providing the person with the information material to his decision, such as the risks and benefits, and it needs to (3) require the participant to render and communicate the decision to participate. A fundamental ethical principle underlying the process relates to autonomy, the participant’s right to decide on research activities that pertain to his/her care, to the nature of participation, and to how he/she will be identified and observed.

#### The ethical principles related to proxy consent

While not currently applicable in Israel, substitute judgment theoretically comes into play in the case of PwDs who cannot provide consent due to their inability to comprehend the information, or to make a decision, or to communicate it. In such cases, there is a need for a proxy, typically a relative or close friend with intimate knowledge of the PwD pre-morbidly, to consider how the PwD would have responded to the invitation to participate in the study. A theoretical and practical consideration that arises is that the substitute judgment exercised by proxies can potentially differ from the way the PwDs actually would have decided for themselves. A further ethical principle is that of beneficence and non-maleficence, which requires the proxy to consider what is best for the participant and to act to minimize any potential harm.

Proxies acting with close personal knowledge of PwDs, and guided by the principle of beneficence, may nevertheless find themselves in situations in which these two points of reference conflict with each other. Stated differently, there can be cases when a substitute decision may lead to a determination different from that reached on the basis of beneficence. There are also times when some proxies exercise neither substitute judgment nor beneficence. In the author’s experience, this is most often the case with legal guardians, who are frequently disinclined to be bothered with issues of research, whether or not the research may provide benefits to their wards. Finally, some PwDs have neither a legal guardian nor a family member or friend who can serve as a proxy. If such PwDs cannot provide consent on their own, they are normally excluded from research that involves significant risks. However, in the U.S., when proposed research holds potential benefits and no more than minimal risks, volunteers have been used to meet with PwDs and make determinations as to whether participation is appropriate based on this acquaintance and on the principle of beneficence.

#### Assent

When the PwD is unable to provide consent, but proxy consent is provided, the researcher is nevertheless responsible for evaluating assent, and acting accordingly. Even though the study may involve little or no risk, such as cases in which the PwDs are being observed or participating in a leisure activity, PwDs can still be bothered by the research procedures or may disagree with participating. It is the duty of the investigator to pay attention to the PwDs’ reaction to research procedures and to withdraw when a PwD appears to be bothered or unwilling to be involved with the procedures.

### Alternative frameworks for handling research with PwDs – The case in the US

The U.S. is among the countries in which family proxies can provide consent for PwDs to be involved in research, provided that an Institutional Review Board (IRB) has approved the research and the method for obtaining such consent. Before approving studies and their procedural safeguards, IRBs weigh the risks and benefits of proposals in order to assure an ethical balance between research needs and protection of participants. The primary regulation governing research with human participants in the U.S. can be found at 45 CFR 46 [26]. It was promulgated by the U.S. Department of Health and Human Services’ Office for Human Research Protections and describes the requirements for IRBs, which are to include at least five members with diverse backgrounds and the expertise to evaluate prospective studies (45 CFR 46.107) [26]. While informed consent is required from human study participants, IRBs can deviate from the consent requirement in specific cases: “An IRB may approve a consent procedure that omits some, or alters some or all, of the elements of informed consent set forth [above] provided the IRB satisfies the requirements of paragraph (f)(3) of this section...” (45 CFR 46.116(f)(2)) [26]. Those requirements are:

(i) The research involves no more than minimal risk to the subjects;

(ii) The research could not practicably be carried out without the requested waiver or alteration;

(iii) If the research involves using identifiable private information or identifiable biospecimens, the research could not practicably be carried out without using such information or biospecimens in an identifiable format;

(iv) The waiver or alteration will not adversely affect the rights and welfare of the subjects; and.

(v) Whenever appropriate, the subjects or legally authorized representatives will be provided with additional pertinent information after participation (45 CFR 46.116(f)(3)) [26].

The regulations specifically provide that “[w]hen some or all of the subjects are likely to be vulnerable to coercion or undue influence, such as children, prisoners, individuals with impaired decision-making capacity, or economically or educationally disadvantaged persons, additional safeguards [must] have been included in the study to protect the rights and welfare of these subjects.” (45 CFR 46.111(b)) [26]. In the case of children, consent is required from parents or a guardian, and assent is required from the child as well. In cases in which “the IRB determines that the capability of some or all of the children is so limited that they cannot reasonably be consulted or that the intervention or procedure involved in the research holds out a prospect of direct benefit that is important to the health or well-being of the children and is available only in the context of the research, the assent of the children is not a necessary condition for proceeding with the research.” (45 CFR 46.408) [26]. Other provisions provide guidance concerning research which does not hold out the prospect of direct benefit, but which is likely to yield generalizable knowledge. (45 CFR 46.406) [26].

It is easy to observe the parallel between parents of children and family proxies of persons with advanced dementia. Both commonly make a wide range of day to day decisions, including weighty healthcare decisions on behalf of their loved ones. Thus, permitting consent by family proxies for the participation of PwDs in clinical research in the U.S. is seen as a reasonable extension of functions expected of, and already being carried out by family proxies on behalf of PwDs, and it is common practice for IRBs to proceed accordingly.

The final decisions as to the type of consent and assent required, and any specific limitations, are made by IRBs based on their determination of the study’s risks and benefits to participants, among other considerations (45 CFR 46.116(f)(2)) [26]. In addition to appropriate safeguards, persons from whom consent or proxy consent is sought may withhold or withdraw it at any time (45 CFR 46.116(b)(8)) [26]. Other criteria include the requirement that the proposed trial use procedures that are consistent with sound research design and that do not unnecessarily expose participants to risk (45 CFR 46.111(a)(1)(i)) [26]. If the research questions can be answered by study of populations less vulnerable than children or PwDs the study should not be conducted using a vulnerable sample.

### Research review committees – Other countries

Canada uses methods similar to those in the U.S. to enroll persons with advanced dementia in clinical research, i.e., after a study is approved by an institution’s research ethics board (REB), informed consent can be sought from family members of PwDs in order to allow participation in the study, e.g., [27]. A similar approach governs in Great Britain, where, for example, a recent large trial about approaches to care reported that “[w]ritten consent was provided by next of kin when individuals did not have mental capacity to consent for themselves,” and that “[t]his research was reviewed and approved by the Oxford C National Research Ethics Committee” [28]. The need to accommodate different IRB’s of different institutions, each of which may have its own sense of priorities, can complicate multi-site clinical research on dementia. In multi-national trials this requires adherence to the rules of multiple regulatory bodies [22].

### Advance research directives

One proposed solution to the problem of obtaining informed consent for persons with dementia is the use of “advance research directives” in which persons who have not lost their capacity to give legal consent, may consent to participate in future research [29]. This consent would be given prior to diagnosis, or at the earliest stages of dementia [30, 31]. However, the low rates of utilization of advance directives for health care decisions makes the notion of wide use of advance research directives seem implausible [32].

## Conclusion

Given the extreme importance of research to develop an understanding of the care needs of PwDs, to test alternatives in order to improve care, to examine current care practices, and to identify areas of concern, all with the goal of enhancing the lived experience of PwDs and their caregivers, barriers to such research must be reconsidered and then minimized. Specifically, it is imperative that the Israel Ministry of Health revise Regulatory Procedure 14, and, if necessary, that underlying laws be amended in order to promote such research and to place Israel on par with other countries in handling informed consent for those who have cognitive difficulties. Models which allow proxy consent exist in developed countries, and Israel should use these models as a basis upon which to facilitate the advancement of knowledge in the field of dementia care. This would be the first of numerous steps needed to improve care. Other measures would include increased targeted funding, incentives to universities and dementia services to test models for improved care, and recognizing dementia studies as a specific focus of academic pursuit, as at Bangor University, the University of Stirling, the University of Worcester, and the University of Bradford [33–36]. By acting in these areas, Israel has the potential to join the ranks of other nations in enhancing the lives of persons with dementia and their caregivers.

## Abbreviations

ADL: Activities of Daily Living
IRB: Institutional Review Board
PwD: Person with Dementia
PwDs: Persons with Dementia
REB: Research Ethics Board
